# Supplementary material for: MRI- and CT-determined changes of dysphagia / aspiration-related structures (DARS) during and after radiotherapy
Source: PLoS One. 2020 Sep 2;15(9):e0237501. doi: 10.1371/journal.pone.0237501 (PMC7467287; doi:10.1371/journal.pone.0237501)
Supplement: S1 File — (DOCX) [file pone.0237501.s002.docx]

**List of Abbreviations**

ADI-D Anderson Dysphagia Inventory – D (Deutsch – German)

ART Adaptive Radiotherapy

CT Computertomography

CUP Cancer of Unknown Primary

DARS Dysphagia / Aspiration related Structures

D_mean_ mean Dose

D_max_ maximum Dose

DSI Dysphonia Severity Index

DVH Dose Volume Histogram

EORTC European Organization for Research and Treatment of Cancer

ESCALOX Do Selective Radiation Dose Escalation and Tumour Hypoxia Status Impact the Loco-regional Tumour Control after Radio-chemotherapy of Head & Neck Tumours?

FEES Fiber Optic Evaluation of Swallowing

FMISO Fluoromisonidazol

FOIS Functional Oral Intake Scale

Gy Gray

GTV Gross Tumor Volume

H&N Head and Neck Cancer

IGRT Image Guided Radiotherapy

IMRT Intensity Modulated Radiotherapy

MRgRT Magnetic Resonance Imaging Guided Radiotherapy

MRI Magnetic Resonance Imaging

PRO Patient Reported Outcome

PTV Planning Target Volume

QoL Quality of Life

SIB Simultaneous Integrated Boost

SWOAR Swallowing Organs at Risk

TUM Technical University of Munich

RT Radiotherapy
