# Supplementary material for: MRI- and CT-determined changes of dysphagia / aspiration-related structures (DARS) during and after radiotherapy
Source: PLoS One. 2020 Sep 2;15(9):e0237501. doi: 10.1371/journal.pone.0237501 (PMC7467287; doi:10.1371/journal.pone.0237501)
Supplement: S1 Table — (DOCX) [file pone.0237501.s003.docx]

| Cumulative value  ADI-D | Definition [translated into English] |
| --- | --- |
| <55 | Sure [conspicuous](https://dict.leo.org/englisch-deutsch/conspicuous) |
| 55 - 70 | Rather conspicuous |
| >70 | Rather inconspicuous |

**S1 Table.** Grading of ADI-D
