# Supplementary material for: MRI- and CT-determined changes of dysphagia / aspiration-related structures (DARS) during and after radiotherapy
Source: PLoS One. 2020 Sep 2;15(9):e0237501. doi: 10.1371/journal.pone.0237501 (PMC7467287; doi:10.1371/journal.pone.0237501)
Supplement: S2 Table — (DOCX) [file pone.0237501.s004.docx]

| **Grad** | **Graduation** |
| --- | --- |
| **1** | Material does not penetrate into the airways. |
| **2** | Material penetrates into the airways, is located cranial of the glottis and will be ejected. |
| **3** | Material penetrates into the airways, is located cranial of the glottis and will be not ejected |
| **4** | Material penetrates into the airways, has contact to the glottis and will be ejected |
| **5** | Material penetrates into the airways, is located cranial of the glottis and will be not ejected |
| **6** | Material penetrates into the airways, is located caudal of the glottis and will be ejected into the larynx or out of the airways. |
| **7** | Material penetrates into the airways, penetrates caudal of the glottis, will be not ejected from the trachea despites effort |
| **8** | Material penetrates into the airways, penetrates caudal of the glottis, there is no effort to eject the material from the trachea |

**S2 Table.** 8-point-Penetrations-Aspirations-Scale (PAS) by Rosenbek [13]
