# Supplementary material for: MRI- and CT-determined changes of dysphagia / aspiration-related structures (DARS) during and after radiotherapy
Source: PLoS One. 2020 Sep 2;15(9):e0237501. doi: 10.1371/journal.pone.0237501 (PMC7467287; doi:10.1371/journal.pone.0237501)
Supplement: S3 Table — (DOCX) [file pone.0237501.s005.docx]

| Level | Need of tube feeding (Level 1-3) |
| --- | --- |
| 1 | Tube feeding – no oral food intake |
| 2 | Tube feeding with minimal / inconsistent oral food and / or liquid intake |
| 3 | Tube feeding with concomitant oral food and liquid intake |
|  | Complete oral nutrition (Level 4-7) |
| 4 | Complete oral nutrition with one consistency |
| 5 | Complete oral nutrition of all consistence but with special preparation (oral stimulation, special positioning of food in the mouth, chopped pieces) or compensation (support for chewing, control of jaws, use of instruments |
| 6 | Complete oral nutrition of all consistency without special preparation, but with limitation of food(defined amount, avoidance of food with problematic consistency |
| 7 | Complete oral nutrition without limitation |

**S3 Table.** Scale of oral food intake (FOIS) by Crary et al. [15]
