# Supplementary material for: MRI- and CT-determined changes of dysphagia / aspiration-related structures (DARS) during and after radiotherapy
Source: PLoS One. 2020 Sep 2;15(9):e0237501. doi: 10.1371/journal.pone.0237501 (PMC7467287; doi:10.1371/journal.pone.0237501)
Supplement: S4 Table — (DOCX) [file pone.0237501.s006.docx]

| DSI-limits | Severity of Dysphonia |
| --- | --- |
| > 4,2 | 0 = normal |
| 4,2 - > 1,8 | 1 = low grade |
| 1,8 - > –1,2 | 2 = moderate |
| < –1,2 | 3 = high grade |

S4 Table. Classification of dysphonia by using DSI-values defined by Wyuts [23]
