# Supplementary material for: MRI- and CT-determined changes of dysphagia / aspiration-related structures (DARS) during and after radiotherapy
Source: PLoS One. 2020 Sep 2;15(9):e0237501. doi: 10.1371/journal.pone.0237501 (PMC7467287; doi:10.1371/journal.pone.0237501)
Supplement: Attachment — Submitted filename: Response_to_Reviewers.docx [file pone.0237501.s007.docx]

**Response to the editor and reviewers by Dr. Steffi Pigorsch**

**Concering:**

PONE-D-19-32133
MRI-determined Changes of Dysphagia / Aspiration related Structures (DARS) during and after Radiotherapy: influence on Function and Quality of Life (QoL) of Head and Neck Cancer Patients
PLOS ONE

Dear Dr. Kessel, 

Thank you for submitting your manuscript to PLOS ONE. After careful consideration, we feel that it has merit but does not fully meet PLOS ONE’s publication criteria as it currently stands. Therefore, we invite you to submit a revised version of the manuscript that addresses the points raised during the review process.

**Note**: We changed the corresponding author From Dr. Kerstin Kessel to Dr. Steffi Pigorsch.

**Dear Editor and dear Reviewers,**

We thank you kindly for your detailed and helpful comments. As recommended by the third reviewer, we have subjected the manuscript to a complete and thorough revision and verification. Of course, we would be happy to make our anonymized raw data available. Furthermore, we have elaborated on our procedure of patient selection for the study in question. The entire statistical analysis has once again been evaluated by two statisticians, while a native speaker with a language training background proof-read and corrected the manuscript. All abbreviations have been set in parentheses following the initial mention of the respective terminology.

Your´s sincerely, Dr. Steffi Pigorsch

==============================
ACADEMIC EDITOR: The study is interesting. Please kindly respond to the questions raised by the reviewers.

==============================

We would appreciate receiving your revised manuscript by Feb 13 2020 11:59PM. When you are ready to submit your revision, log on to <https://www.editorialmanager.com/pone/> and select the 'Submissions Needing Revision' folder to locate your manuscript file.

**NOTE:** Please note, we thank You very much for extension of deadline until March 20^th^ 2020.

 Additional Editor Comments (if provided):

The study is interesting. Please kindly respond to the questions raised by the reviewers.

Journal Requirements:

When submitting your revision, we need you to address these additional requirements.

1. Please ensure that your manuscript meets PLOS ONE's style requirements, including those for file naming. The PLOS ONE style templates can be found at

<http://www.journals.plos.org/plosone/s/file?id=wjVg/PLOSOne_formatting_sample_main_body.pdf> and <http://www.journals.plos.org/plosone/s/file?id=ba62/PLOSOne_formatting_sample_title_authors_affiliations.pdf>

**Answer:** We followed these recommendations.

2. Please note that PLOS journals require authors to make all data underlying the findings described in their manuscript fully available without restriction at the time of publication. When specific legal or ethical requirements prohibit public sharing of a dataset, authors must indicate how researchers may obtain access to the data. PLOS journals will not consider manuscripts for which the following factors influence ability to share data:

- Authors will not share data because of personal interests, such as patents or potential future publications.

- The conclusions depend solely on the analysis of proprietary data, whether these data are owned by the authors, by their funders or institutions, or by other parties.

Therefore, please update your Data Availability statement to indicate how other researchers may gain access to the underlying data reported in the manuscript. For more information, please see: <https://journals.plos.org/plosone/s/data-availability>.

**Answer:** We will make data available in anonymized form as separate file.

3. Please provide additional details regarding participant consent. In the ethics statement in the manuscript, you have specified that 'informed consent was obtained from all individual participants included in the study'. Please clarify whether informed consent was obtained from 17 patients to use their medical records in the retrospective study, or whether consent was obtained from the 5 patients to participate in the prospective study. If informed consent was obtained for both parts of the study, please specify this in both the ethics statement in the manuscript and the online submission form.

If consent was only obtained for 5/17 patients for the prospective part of the study, please clarify whether the patient records of the remaining 12/17 patients were analyzed anonymously, or whether consent was waived by the ethics committee.
 
Additionally, in the ethics statement in the Methods and online submission information, please ensure that you have specified what type of consent you obtained (for instance, written or verbal, and if verbal, how it was documented and witnessed). If your study included minors, state whether you obtained consent from parents or guardians.

**Answer:** We have explained in detail the ethics statement and the patient information as well as the procedure for patient informed consent in the Methods section.

 4. To comply with PLOS ONE submission guidelines, in your Methods section, please provide additional information regarding your statistical analyses. For more information on PLOS ONE's expectations for statistical reporting, please see <https://journals.plos.org/plosone/s/submission-guidelines.#loc-statistical-reporting>.

**Answer:** We have fully complied with the submission guidelines and affirm that statistical analyses have been performed by the responsible statistician and cross-checked by yet another statistician.

5. At this time, we ask that you please clarify whether the 'Ethics Committee of the Medical Faculty TUM' specifically reviewed and approved both the retrospective and prospective parts of the present study.

**Answer:**  Firstly, the Ethics Committee of the Medical Faculty TUM reviewed and approved the retrospective part of the study. The prospective part of the study was reviewed and proved as well by the Ethics Committee of the Medical Faculty TUM with an amended version of the initial trial protocol.

6. In your Methods section, please provide additional information about the participant recruitment method and the demographic details of your participants used in the prospective study. Please ensure you have provided sufficient details to replicate the analyses such as: a) the recruitment date range (month and year), b) a description of any inclusion/exclusion criteria that were applied to participant recruitment, c) a description of how participants were recruited, and d) descriptions of where participants were recruited and where the research took place.

**Answer:** We have thoroughly covered the questions raised in the Methods section.

 7. Please include additional information regarding the questionnaires (ADI-D, Voice-H-I, EORTC QLQ-H&N35 Mean) used in the study and ensure that you have provided sufficient details that others could replicate the analyses. For instance, if you developed a questionnaire as part of this study and it is not under a copyright more restrictive than CC-BY, please include a copy, in both the original language and English, as Supporting Information.

**Answer:** All questionnaires are standardized and will be uploaded as a supplement. At no time was a questionnaire of our own making employed in the study.

Reviewers' comments:

Reviewer's Responses to Questions

**NOTE:** Please note, our answers are included in yellow.

1. Is the manuscript technically sound, and do the data support the conclusions?

The manuscript must describe a technically sound piece of scientific research with data that supports the conclusions. Experiments must have been conducted rigorously, with appropriate controls, replication, and sample sizes. The conclusions must be drawn appropriately based on the data presented.

Reviewer #1: No

Reviewer #2: Yes

Reviewer #3: No

**Answer:** We have subjected the manuscript to a complete and thorough revision and verification as recommended by the third reviewer.

2. Has the statistical analysis been performed appropriately and rigorously?

Reviewer #1: No

Reviewer #2: I Don't Know

Reviewer #3: No

**Answer:** The complete statistical analysis has been revised anew by two statisticians.

3. Have the authors made all data underlying the findings in their manuscript fully available?

The [PLOS Data policy](http://www.plosone.org/static/policies.action#sharing) requires authors to make all data underlying the findings described in their manuscript fully available without restriction, with rare exception (please refer to the Data Availability Statement in the manuscript PDF file). The data should be provided as part of the manuscript or its supporting information, or deposited to a public repository. For example, in addition to summary statistics, the data points behind means, medians and variance measures should be available. If there are restrictions on publicly sharing data—e.g. participant privacy or use of data from a third party—those must be specified.

Reviewer #1: Yes

Reviewer #2: Yes

Reviewer #3: No

**Answer:** We will, of course, make our anonymized raw data available.

 4. Is the manuscript presented in an intelligible fashion and written in standard English?

PLOS ONE does not copyedit accepted manuscripts, so the language in submitted articles must be clear, correct, and unambiguous. Any typographical or grammatical errors should be corrected at revision, so please note any specific errors here.

Reviewer #1: Yes

Reviewer #2: Yes

Reviewer #3: No

**Answer:** The manuscript has been corrected by a native speaker with a language training background.

5. Review Comments to the Author

Please use the space provided to explain your answers to the questions above. You may also include additional comments for the author, including concerns about dual publication, research ethics, or publication ethics. (Please upload your review as an attachment if it exceeds 20,000 characters)

Reviewer #1: The authors performed a retrospective study of 17 patients with head and neck cancers to investigate the usefulness of MRI before, during and after radiation therapy.

Although the focus of this paper seems to be important in future radiotherapy, this paper has some serious problems.
First of all, there were only 17 cases in this study. In addition, this study included tumors at various sites and stages. There were also various treatments for tumors. The possibility that these tumor sites and treatments affected dysphagia, dysphonia, or other symptoms cannot be ruled out, and it is difficult to say that the scientific validity of the results of this study is guaranteed.
The authors should increase the number of cases and unify the tumor site and treatment.

**Answer:** As the discussion indicates, there are only very few studies on this topic comprising a larger number of patients. All in all, this patient collective largely proves to be non-compliant. Not all patients are willing to tolerate the considerable amount of expenditure involved in conducting the necessary examinations. The small number of 17 retrospective patients is due to the required entirety of DARS imaging. Only patients with complete DARS imaging were included. All patients still alive at the time of the prospective study were invited. However, only five of these patients agreed to participate in the evaluation. We plan to carry out further swallowing examinations in a prospective follow-up study.

Other remarks are provided below.

1) The introduction should be a little shorter. For some sentences, the authors should move them to the discussion part.

**Answer:** We have revised and shortened the Introduction section.

2) I think that abbreviations should be unified. Please consider whether to write abbreviations in the text or at the end of the manuscript.

- All abbreviations have been named and set in parentheses after the first use of the term.

  3) The authors should unify the description of date. “1.1.2008” or “5/2012”

**Answer:** All date descriptions have been uniformed.

Reviewer #2: This is a well written paper regarding MRI-based evaluation of Dysphagia/Aspiration Related Stractures(DARS).
Among 780 patients with head and neck cancer, 17 patients who had MRI before RT (MRI 1), at 40Gy (MRI 2) and 6 weeks after RT (MRI 3) were selected and retrospectively analyzed after the DARS were delineated on MRI and CT.
Furthermore, as a prospective part of the study, the authors carried out voice and swallowing tests in five of the 17 patients.
As the results, they found statistically significant changes in volume of four DARS; including increases and a decrease in volume depending on the sub-component of the DARS. However, there was no dose dependence of total dose to DARS in the alternation of volumes.
From the symptom described on the patient charts, dysphagia was related to the mean dose of DARS, but there was no significant relationship between volume changes of DARS and dysphagia.

Impression:
The authors investigated well in anatomical and functional changes related to DARS. However, they seemed to fail in constructing a definite conclusion from the findings they observed, although each finding is valuable itself. It will be acceptable for publication if the authors describes more of other related studies regarding this theme, and make it clear that what’s new this study(studies) could be added to the current shared knowledges.

**Answer:** In the last two years, other studies on this subject were compiled in various reviews due to the fact that the individual studies comprised only a small number of patients. These investigations were examined in detail in both the review and the individual publications.

Questions:
How the author selected 5 patients who participate in prospective study from the 17 patients?

**Answer:** All surviving patients at the time of our prospective study were invited. However, only five agreed to participate in the evaluation.

Minor points
Page 4, paragraph 2, last line: A full stop (and perhaps some preceding words) is missing.

**Answer:** The manuscript has been fully revised as recommended by Reviewer 3.

Reviewer #3: The goal of the authors of the manuscript is to the potential of MR-image guidance for RT of head and neck tumors because of better discrimination and resolution of soft tissues as DARS in MRI compared to CT.

The manuscript is poorly written and the authors do not respect the basic rules of scientific writing. Some sentences are impossible to understand due to the apparent absence of knowledge of some rules of english writing

**Answer:** We have taken this criticism to heart and implemented necessary changes and improvements in both our general and scientific style of writing. We have been careful to observe English language standards pertaining to correct spelling, punctuation, and common usage.

The authors are not consistent in their use of acronyms. acronyms must be defined first completely in english with acronyms written between parenthesess after the definition

**Answer:** We have ensured a thorough revision concerning this point as well.

In a scientific paper the authors must always assume that their readers do not know fully the content of the paper and thus must be crystal clear. They must "take the readers gently by the hand" to present their scientific goal, to explain the materials and methods used, to justify the statistical methods used, to display clearly their data and then discuss seriously theirs results and especially the limitations of their study if there are any.

**Answer:** We can affirm that we are well aware of this goal and have adapted our manuscript accordingly so that its contents can be fully understood and appreciated by non-professionals as well.

1) This abstract is unacceptable full of acronyms not previously defined

**Answer:** We would like to politely call to your attention that a table of acronyms can be found as a supplementary file.

The entire content has not only been adapted to all PlosOne requirements, but completely revised as well.

Integration of MRI into RT-planning gives the opportunity to delineate DARS more precisely
for RT. DARS are important for QoL in H&N patients since late effects cause dysfunction. By
MRgRT, (comma) improved DARS-contouring and adaption during RT is possible. 17 H&N-patients
(treated 5/2012 - 8/2015) were analyzed retrospectively. 14/17 had concomitant chemotherapy.
Median time RT- phoniatric evaluation was 1.78 years [range 0.42-4.26]. Patients were treated
by IMRT median single dose: 2Gy [1.7-2.2Gy] and cumulative dose: 70Gy [64-70.4Gy]. DARS
were delineated on MRI and planning CT scans and co-registered with all RT plans. 5/17
patients participated in a prospective voice and swallowing test (last RT – examination: 22.2
months (average)). For PRO Anderson Dysphagia Inventory, Voice Handicap Index and
EORTC QLQ- H&N 35 were applied.
FEES, voice test and automatic voice processing were used for objective assessment.
There was neither a dose dependence of Dmean DARS volume-changes over time nor of
dysphonia and no correlation between volume changes, dysphagia or dysphonia. One additional
Gy on Dmean DARS causes a 7.5fold risk to suffer from early (first 6 months after end of RT)
dysphagia and 4.7fold later than 6 months. By FEES 3/5 patients were diagnosed with post
radiogenic changes of morphology and 4/5 with reduced sensitivity. Functional swallowing test
detected disturbances in all cases. Dysphonia had a high variation. Swallowing related QoL
was "rather conspicuous". Patients ranked themselves good in EORTC QLQ-H & N35.
Every additional Gy on Dmean of DARS increases the risk for late dysphagia and should be
avoided.

2) It is not acceptable that the authors do not make available extensively their data about patients for so-called ethical reasons. The anonymisation of patients is always possible and garantee the private life and the identity of all patients

**Answer:** All data will be made accessible as anonymized raw data.

3) It is not clear how the 17 patients includec in this study were chosen out of more 700 patients

**Answer:** The precise method pertaining to the choice of patients from a total number of 780 patients was clearly explained and can be found in the Methods section.

4) The introduction is very poor

**Answer:** The Introduction has been revised in its entirety.

5) the Materials and Methods are not clear at all. There is none justification for any of the statistical method used. A Specialist in Biostatistics should be included among the authors

**Answer:** A statistician is amongst the authors. Moreover, the data has since been examined twice by other statisticians. Early on, the presented analysis had already been evaluated by our statistician.

6) The inclusion of various tumors at different stages with very few patients for each stage is a big flaw of this study

**Answer:** This is certainly the case. Patient selection for this study is limited, because complete MRI imaging of DARS is a prerequisite and vital criterion for inclusion in the study. MRIs were not performed for the purpose of DARS imaging, but for reasons of portrayal of the tumor and lymph nodes with the aim of contouring the target volume for radiation therapy. This explains the heterogeneity of the examined tumor patients.

7) The classification of the tumors is not explicit for any common reader

**Answer:** For details and explanation of tumor classification, we recommend further reading at ww.UICC.org.

8) The presentation of the results is very confusing

**Answer:** The presentation of the results has been exhaustively restructured.

9) the discussion must be completely be rewritten

**Answer:** Both the discussion and the entire paper have been rewritten. We thank you kindly for your valuable comments.

A dramatic revision is mandatory

**Answer:** This has indeed been implemented.

6. PLOS authors have the option to publish the peer review history of their article ([what does this mean?](https://journals.plos.org/plosone/s/editorial-and-peer-review-process#loc-peer-review-history)). If published, this will include your full peer review and any attached files.

If you choose “no”, your identity will remain anonymous but your review may still be made public.

**Answer:** We want to publish the whole history of review.

**Do you want your identity to be public for this peer review?** For information about this choice, including consent withdrawal, please see our [Privacy Policy](https://www.plos.org/privacy-policy).

Reviewer #1: Yes: Eiichiro Okazaki

Reviewer #2: No

Reviewer #3: No
