# Supplementary material for: MRI- and CT-determined changes of dysphagia / aspiration-related structures (DARS) during and after radiotherapy
Source: PLoS One. 2020 Sep 2;15(9):e0237501. doi: 10.1371/journal.pone.0237501 (PMC7467287; doi:10.1371/journal.pone.0237501)
Supplement: Attachment — Submitted filename: Rebuttal_Letter_PlosOne_Pigorsch_20200609.doc [file pone.0237501.s008.doc]

**Klinikum rechts der Isar
Public Law Institution**

**Department of RadioOncology and Radiotherapy**

Member of

**Univ.-Prof.**

**Dr. med. S. E. Combs**

Head of Department

Ismaninger Straße 22
81675	München
E-Mail: direktion.radonk@mri.tum.de
Tel: 089 4140 - 4501 / 4502
Fax: 089 4140 - 4477

www.radonc.med.tu-muenchen.de

Board of Directors:

Professor M. Schwaiger, MD

(Medical Director, Chairman)

Dr. E. Frank

(Commercial Director)

S. Großmann

(Director of Nursing)

Professor B. Hemmer, MD

(Dean)

Bank account:

Bayer. Landesbank Girozentrale

BIC: BYLADEMM

IBAN: DE82 7005 0000 0000 0202 72

VAT ID: DE 129 52 3996

Jason CH Hsieh, M.D. Ph.D
Academic Editor
PLOS ONE

Klinikum rechts der Isar · MST · 81664 München

Munich, 2020/06/30.11.2026

**Rebuttal Letter on PONE-D-19-32133R1**

Dear Dr Hsieh, dear colleagues,

Please find attached our answers to the questions and concerns of the reviewers to our re-submitted manuscript.

Thank You for Your support.

Yours sincerely,

Prof. Dr. med. S.E. Combs	Dr.med. St. U. Pigorsch

Head of Department	Managing Senior Physician

**Comments to the Author**

1. If the authors have adequately addressed your comments raised in a previous round of review and you feel that this manuscript is now acceptable for publication, you may indicate that here to bypass the “Comments to the Author” section, enter your conflict of interest statement in the “Confidential to Editor” section, and submit your "Accept" recommendation.

Reviewer #1: All comments have been addressed

Reviewer #2: All comments have been addressed

Reviewer #3: (No Response)

*Answer: We would like to center Your attention to above reviewer comments.*

2. Is the manuscript technically sound, and do the data support the conclusions?

The manuscript must describe a technically sound piece of scientific research with data that supports the conclusions. Experiments must have been conducted rigorously, with appropriate controls, replication, and sample sizes. The conclusions must be drawn appropriately based on the data presented.

Reviewer #1: No

Reviewer #2: Yes

Reviewer #3: No

***Answer:*** *For further explanation please see our answer to each reviewer comment.*

3. Has the statistical analysis been performed appropriately and rigorously?

Reviewer #1: No

Reviewer #2: Yes

Reviewer #3: No

***Answer:***  *We refer to our response to the reviewer comments upon submission of the revised manuscript: “The complete statistical analysis has been revised anew by two statisticians.”*

*We must emphasize the status of our second statistician, who was independent and not involved in the initial analysis. This data review and statistics check was done especially in light of the reviewer comments.*

4. Have the authors made all data underlying the findings in their manuscript fully available?

The [PLOS Data policy](http://www.plosone.org/static/policies.action#sharing) requires authors to make all data underlying the findings described in their manuscript fully available without restriction, with rare exception (please refer to the Data Availability Statement in the manuscript PDF file). The data should be provided as part of the manuscript or its supporting information, or deposited to a public repository. For example, in addition to summary statistics, the data points behind means, medians and variance measures should be available. If there are restrictions on publicly sharing data—e.g. participant privacy or use of data from a third party—those must be specified.

Reviewer #1: Yes

Reviewer #2: Yes

Reviewer #3: Yes

5. Is the manuscript presented in an intelligible fashion and written in standard English?

PLOS ONE does not copyedit accepted manuscripts, so the language in submitted articles must be clear, correct, and unambiguous. Any typographical or grammatical errors should be corrected at revision, so please note any specific errors here.

Reviewer #1: Yes

Reviewer #2: Yes

Reviewer #3: Yes

6. Review Comments to the Author

Please use the space provided to explain your answers to the questions above. You may also include additional comments for the author, including concerns about dual publication, research ethics, or publication ethics. (Please upload your review as an attachment if it exceeds 20,000 characters)

Reviewer #1: I confirmed the revised paper and the authors' response to the reviewer's comments.

I pointed out that the number of subjects in this study was very small (17 patients), which is not enough to obtain significant results. I think the authors' response to this indication is reasonable. Accumulation of a sufficient number of cases requires a lot of labor and cost. However, this fact does not make up for the small number of cases, and I think that the fact that sufficient cases have not been obtained in statistical studies is a huge disadvantage. I believe that the authors' responses are not sufficient answers regarding this statistical disadvantage. If there is not sufficient statistical proof, I recommend increasing the number of cases or writing a paper in the form of a case report.

***Answer:*** *Neither labor nor the cost of an extended study were the reasons for the limited patient number. We explained in detail how the number of 17 patients was reached. MR scans representing all DARS completely at each point of time were our primary focus. Unfortunately, we were able to only extract 17 patients from a total of 780 with this inclusion criteria. The follow-up data on DARS organ function of these 17 patients were gathered from patient charts retrospectively.*

*Retrospective analysis in general are impaired by availability of complete datasets or images. We have referred to this limitation and the constricted conclusions. The findings of the five prospective examined patients for their swallowing function after therapy were only statistically described.*

*However, the main part of the manuscript deals with the 17 retrospective patients investigated for their DARS organ changes over a longer course of time including radiotherapy treatment weeks and follow-up time.*

Reviewer #2: (No Response)

Reviewer #3: The authors have made significant improvements to their manuscript and provided transparently their data.
Nevertheless, The number of quite different patients at different stages of their tumors enrolled in this study is a major flaw of this investigation.

***Answer:*** *The presented data was generated from a retrospective analysis focusing on morphologic and volumetric changes of swallowing structures over the course of radiotherapy and later during follow-up by MR and planning CT scans. One goal was to improve the delineation of DARS by co-registration of MR scans to the planning CT scans for better anatomic discrimination. The other aim was to demonstrate and prove the hypothesis that DARS were changed over time by means of radiotherapy in regards to volume and structure. The last point – the influence of radiotherapy dose to the amount of DARS volume and function changes - could not be proven safely because only 17 of the investigated patients fulfilled the inclusion criteria.*

*If a prospective study for DARS evaluation were designed, the inclusion criteria would be: tumor entity, tumor stage, treatment intention (definitive or postoperative radiotherapy), combination with systemic therapy, imaging: CT and MR scans at defined time points, base line, and follow-up investigation of swallowing and voice function.*

The title and goal of this manuscript is "MRI-determined Changes of Dysphagia / Aspiration-Related Structures (DARS) during and after Radiotherapy: Influence on Function and Quality of Life (QoL) of Head and Neck Cancer Patients". This study was supposed to support the hypothesis according to which Function and Quality of Life (QoL) of Head and Neck Cancer Patients are significantly improved. This goal could not be reached with such a very low number of patients who participated to this study.

***Answer:*** *Improvement of QoL for HNC patients was not the goal of this study.*

*The aims of our study were clearly defined at the conclusion of the INTRODUCTION: “The aim of this work was to assess the changes of DARS during and after radiooncological treatment of HNC by co-registration of static MRI and planning CT scans for better delineation of DARS in order to detect morphological changes over the course of RT. The second aim was to compare these dosimetric and volumetric data to results of functional investigation of swallowing and voice.”*

***The title of the submitted manuscript is obviously misleading for the reviewer. We like to apologize for this. To focus on the main part of the investigation we changed the title.***

***From: "MRI-determined Changes of Dysphagia / Aspiration-Related Structures (DARS) during and after Radiotherapy: Influence on Function and Quality of Life (QoL) of Head and Neck Cancer Patients".***

***To: "MRI- and CT-determined Changes of Dysphagia / Aspiration-Related Structures (DARS) during and after Radiotherapy of Head and Neck Cancer".***

*For our explanation concerning the number of patients, please see above.*

The co-registration of static MR to planning CT scans, made possible a better delineation of DARS than with CT imaging alone. Over the course of RT and posttreatment of HNC, an increase in DARS volume was detected. This result could be very easily expected. The retrospective investigations of DARS of 17 HNC patients contoured on static MRI and co-registered to CT-based RT plans were complemented by prospective clinical swallowing and voice examinations. Unfortunately, only five patients could be investigated prospectively from this group. This small sample size rendered it impossible to safely estimate dose-function effects. **This study does add any groundbreaking information to the field of clinical and basic oncology.**

***Answer:*** *For our explanation concerning the number of patients, please see above.*

7. PLOS authors have the option to publish the peer review history of their article ([what does this mean?](https://journals.plos.org/plosone/s/editorial-and-peer-review-process#loc-peer-review-history)). If published, this will include your full peer review and any attached files.


If you choose “no”, your identity will remain anonymous but your review may still be made public.


**Do you want your identity to be public for this peer review?** For information about this choice, including consent withdrawal, please see our [Privacy Policy](https://www.plos.org/privacy-policy).

Reviewer #1: Yes: Eiichiro Okazaki

Reviewer #2: No

Reviewer #3: No
